# TDP-43 and ubiquitinated cytoplasmic aggregates in sporadic ALS are low frequency and widely distributed in the lower motor neuron columns independent of disease spread

**DOI:** 10.3109/17482961003602363

**Published:** 2010-03-12

**Authors:** Aaron Bodansky, Jae Mun ‘Hugo’ Kim, Lynne Tempest, Amit Velagapudi, Ryan Libby, John Ravits

**Affiliations:** ^a^ Translational Research Program, Benaroya Research Institute at Virginia Mason, Seattle, Washington, USA

**Keywords:** Sporadic ALS, neuropathology, immunohistochemistry (IHC), TDP-43, ubiquitin, cytoplasmic inclusions

## Abstract

Ubiqitinated and TDP-43 immunoreactive cytoplasmic aggregates are hallmark features of ALS molecular pathology. Since clinically most ALS begins focally and advances contiguously, it is important to characterize their distribution. Our objective was to determine the extent and distribution of TDP-43 immunoreactive aggregates in the lower motor neuron columns as a function of disease onset, and to correlate ubiquitinated with TDP-43 aggregates in the lumbar region. We examined TDP-43 cytoplasmic aggregates at four separate neuraxis levels – hypoglossal nucleus and cervical, thoracic, and lumbar anterior horns – in five controls and 20 sporadic ALS nervous systems from patients whose disease began in various sites, i.e. five bulbar, five arm, five trunk, and five leg onsets. We correlated ubiquitinated to TDP-43 aggregates on adjacent histological sections for the lumbar regions. We found that TDP-43 cytoplasmic aggregates are seen in about 8% of motor neurons but there is marked variability between nervous systems, ranging from 0.4% to 20.6%. The aggregates are uniformly distributed within individual nervous systems. There is no obvious correlation between site of disease onset and rate of spread. Almost all ubiquitinated aggregates correlate to TDP-43 aggregates. Thus, TDP-43 immunoreactive cytoplasmic aggregates have a low overall average frequency that does not correlate with either disease course or clinical spread and is the prime ubiquitinated protein.

## Introduction

In 1988, ubiquitinated cytoplasmic aggregates were identified as being distinctive of amyotrophic lateral sclerosis (ALS) neuropathology (1). The cytoplasmic aggregates were seen to exist in two main morphologies, skeins and dense round inclusions. In 2006, TAR DNA-binding protein 43 (TDP-43), a nucleotide binding protein normally located in the nucleus, was found to be the prime ubiquitinated protein in ALS and to mislocalize to and aggregate in the cytoplasm (2,3). TDP-43 cytoplasmic aggregates have been clearly identified in sporadic ALS (SALS) and non-SOD1 familial ALS (FALS) (4,5). Recent studies have also indicated that TDP-43 abnormalities exist in a wide range of other neurodegenerative diseases including frontotemporal lobar dementia with ubiquitinated pathology, corticobasal degeneration, Alzheimer's disease, Parkinson's disease, Huntington's disease, and hippocampal sclerosis. Primary mutations in the *TARDBP* gene that encodes TDP-43 have been identified in 3% of FALS and 1–5% of SALS (6–8), indicating that disturbance of this protein itself may result in disease. For a variety of reasons including the genetic locations of known mutations and the emerging biochemistry of the protein, it is increasingly believed that the toxic property resides in the C-terminus of the protein (9). Recent evidence suggests that the protein's toxicity may be related to soluble rather than aggregated insoluble protein (10,11).

ALS has a focal onset and an orderly contiguous outward spread that creates pathological changes in the nervous system often related to the onset point, at least in terms of neuron counts (12). Since quantitative analysis of frequency and distribution of TDP-43 aggregates in the lower motor neuron columns has not been well studied nor the possible relationship to disease spread defined, we investigated this at four separate neuraxis levels in 20 ALS nervous systems from patients whose disease began at various sites. We find that TDP-43 aggregates are uniformly distributed within individual nervous systems but there is marked variation between nervous systems. We took advantage of our approach to also quantitatively correlate ubiquitinated and TDP-43 aggregates. We find that nearly all ubiquitinated aggregates correlate to TDP-43 aggregates.

## Materials and methods

### Tissue selection

We studied 20 sporadic ALS and five controls nervous systems in our ALS tissue repository. ALS nervous systems were from patients who met modified El Escorial criteria for probable or definite sporadic ALS (13). The nervous systems were chosen to capture the anatomic regions of onset with enough replication (*n* = 5) to obtain semi-quantitative analysis rather than to reflect typical clinical presentations. Accordingly, there were four groups of five based on location of the onset of clinical disease during life – leg, trunk, arm, and bulbar. From each nervous system we studied two sets of paired adjacent, formalin-fixed, paraffin-embedded, 6-μm-thick histological sections from each of the four major neuraxis levels – hypoglossal nucleus and lumbar, thoracic, and cervical anterior horns – for a total of 16 sections from each patient. The two sets of paired adjacent sections were separated by 150–250 μm within each neuraxis level for greater anatomic sampling.

### Immunohistochemistry (IHC)

We performed immunohistochemistry on the paired adjacent sections, one for TDP-43 and one for ubiquitin. The slides were deparaffinized and immersed in Quench endogenous peroxidase for 30 min. Antigen retrieval was performed by applying unmasking solution (VECTOR® Antigen Unmasking Solution no. H-3300, 1:10 dilution) to the slides and then placing them in a Pascal pressure cooker. After washing the slides in 0.1 *M* Tris buffer (pH 7.6), blocking was performed for 5 min using a 0.1 *M* Tris buffer/2% fetal bovine serum (FBS). Both the TDP-43 primary antibody (Proteintech Group Inc. no. 10782-2-AP, rabbit, IgG, polyclonal; dilution 1:2000) and ubiquitin primary antibody (CHEMICON® International no. MAB1510, mouse, IgG, monoclonal; dilution 1:200) were diluted in Tris/FBS solution and applied to their respective sections. The slides were then incubated in a 4°C humidified chamber for 16–20 h. Another round of washing with Tris buffer and blocking in the 0.1 *M* Tris buffer/2% FBS solution was performed prior to secondary antibody application. The secondary antibody (VECTOR® Biotinylated IgG, anti-rabbit for TDP-43 and anti-mouse for ubiquitin BA-9200) in 1:1000 dilution ratio in Tris/FBS buffer, was then applied to both sections and incubated at room temperature for 1 h. After another round of washing in the Tris buffer and blocking in 0.1 *M* Tris buffer/2% FBS, a Vectastain Elite ABC kit (VECTOR® PK-6200) was used on the slides. The slides were rinsed and washed in Tris buffer, and liquid DAB substrate kit (Zymed no. 00-2014) was applied to the slides and incubated at room temperature for 5 min and then washed with distilled water. Slides were counterstained with hematoxylin, dehydrated, cleared, and mounted.

### Digital microscopy

Digital images were taken on a Leica DM 2500 microscope using Spot Advanced software. The procedure was designed to capture the entirety of both anterior horns and hypoglossal nuclei, with maximum resolution. To ensure consistency across the samples, a specific technique was followed during digital microscopy, which varied slightly by neuraxis level due to anatomical differences. A transverse line centered through the central canal divided the anterior and posterior horns. Each anterior horn was then divided into grids that tiled the region of interest and each tile was imaged. The lumbar, cervical, and thoracic sections were all imaged at 400X magnification with grid sizes of 9, 6, and 4 images, respectively; the differences in numbers of images were due to the differences in sizes of the respective anterior horns. The hypoglossal nuclei in the medulla were imaged at 200X magnification and only one picture was needed per motor neuron region. A total of 3554 images at 5.46 pixels/micrometer (for all spinal cord regions) resolution was obtained.

### Data counting

All images were de-identified and given to five separate observers working in ALS histology for counting. The observers received an identical training tutorial instructing them on the counting methodology. This consisted of counting the total number of neurons and the number of neurons showing TDP-43 abnormalities within each image. TDP-43 abnormalities were divided into three categories based on histological morphology – dense round inclusions, skeins, and overlaps of skeins and inclusions. Quantitative assessments of hypoglossal nuclei in the medulla sections were sometimes difficult due to anatomical variation as a function of the exact rostral-caudal level (14). After counting, the data were then re-identified and compiled for statistical analysis.

### Ubiquitin/TDP-43 correlation

For this part of the study, we looked at the lumbar level images. We identified motor neurons with ubiquitin-positive immunostaining and then searched for corresponding TDP-43 immunoreactivity in the paired adjacent section.

### Statistics

We sought correlation between site of onset and distribution of pathology with a repeated measures ANOVA using between-subject factors. The analysis was carried out in R version 2.9.2 (www.r-project.org).

## Results

**Figure 1.**
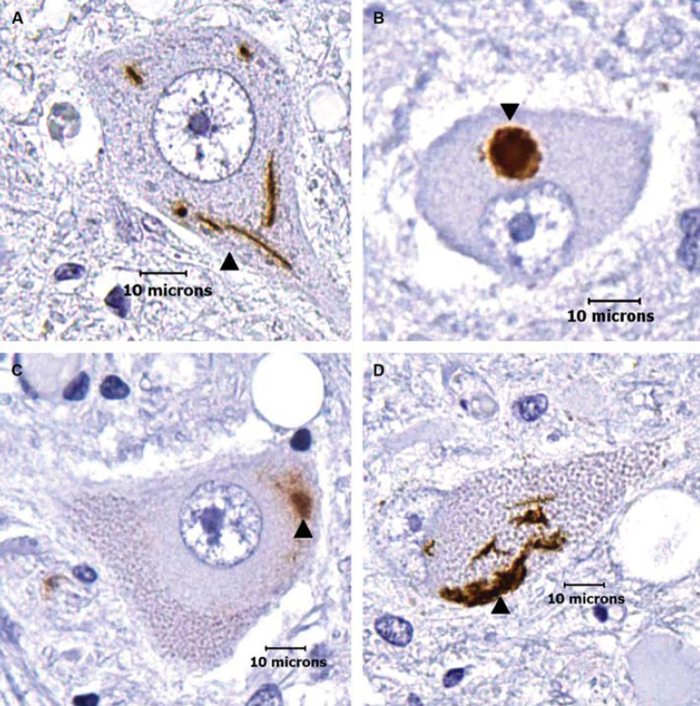
Morphological categories of TDP-43 immunoreactive cytoplasmic aggregates in ALS lower motor neurons. (A) Skeins: these appear as multiple string-like TDP-43-positive aggregates (arrow head). (B) Dense round inclusions: these appear as dense, round TDP-43-positive aggregates (arrow head). (C–D) Overlaps: these appear as an intermediate between skeins and dense round inclusions. In C, the TDP-43-positive aggregate appears similar to a dense round inclusion but has diffuse aggregation emerging from the inclusion body (arrow head). In D, morphology is similar to a skein, but the string-like deposits are locally condensed and form a large, dense deposit (arrow head). Note that the aggregates are seen in what otherwise appear to be morphologically healthy neurons. (Scale bar = 10μm).

TDP-43 immunoreactive cytoplasmic aggregates were seen in all 20 ALS nervous systems (Figure 1). Thirteen nervous systems showed abnormalities at all four levels of the neuraxis, three nervous systems showed abnormalities at three levels, four nervous systems showed abnormalities at two levels, and none of the nervous systems had abnormalities at just one level. Overall, 69 out of 80 levels had at least one abnormality.

TDP-43 immunoreactive cytoplasmic abnormalities were seen in both healthy appearing neurons in ALS nervous systems and in smaller atrophic appearing neurons. In absolute counts, there was an average of 5–6 abnormal neurons per neuraxis level with a range of 0–20. Of the 5345 ALS nervous system neurons that were counted, there was a total of 16 skeins, 154 dense round inclusions, and 278 overlaps of skeins and dense round inclusions (Table I). A total of 448 (8.3%) ALS nervous system neurons had TDP-43 immunoreactive cytoplasmic aggregates. While the overall average amount of motor neurons showing TDP-43 aggregation in all 20 nervous systems was 8.3%, the range between different nervous systems was 0.4–20.6%: four nervous systems showed aggregation in less than 1% of motor neurons and four nervous systems showed aggregation in greater than 15% of motor neurons. Only three of the 1263 (0.24%) control nervous system neurons showed possible immunoreactivity; we retrospectively re-examined these and found they were artifactual and thus indicated background or technical noise in our blinded high-throughput study. While quantitative data regarding nuclear staining in control neurons were not gathered, control neurons seemed to consistently stain for TDP-43 in the nuclei.

**Table I.** Clinical and pathological data.

Row	Onset region	Age*	Gender	Disease duration (years)	CNS ID	Location†	Neuron number‡	Skeins‡	Inclusion‡	Overlaps‡
1	Control	61	Male	NA	7	M	92.25	0	0	0
						C	108.625	0	0	0
						T	35.75	0	0	0
						L	126.5	0	0	0
2	Control	80	Female	NA	19	M	74.375	0	0	0
						C	41.125	0	0.3	0.1
						T	19	0	0	0
						L	78	0	0.1	0
3	Control	77	Male	NA	23	M	75.125	0	0.1	0
						C	50.5	0	0	0
						T	44.625	0	0	0
						L	146.25	0	0.4	0.2
4	Control	57	Male	NA	37	M	49.375	0	0	0
						C	43.875	0	0.7	0.2
						T	17.875	0	0.3	0
						L	62.125	0	0.3	0
5	Control	61	Male	NA	42	M	23.125	0	0	0
						C	60.375	0	0	0.1
						T	18.625	0	0	0
						L	95.5	0	0.1	0
6	Bulbar	81	Male	2	9	M	30.375	0	0.1	0.1
						C	86.125	0.5	6.2	0.6
						T	24.25	0	3.4	1.7
						L	150.625	0.5	11.7	4.8
7	Bulbar	73	Female	1.5	14	M	41.5	0	1.2	0.2
						C	85.5	0.625	3.4	3.7
						T	32.875	0	1.2	1.7
						L	127	0.125	1.8	8.2
8	Bulbar	80	Female	2	18	M	43.75	0	6.8	2.2
						C	100.875	0.375	8	1.9
						T	16.25	0	0.4	0
						L	126.25	0.75	9	4
9	Bulbar	74	Male	3.25	27	M	35.875	0	0	0
						C	75.5	0	0	1.7
						T	15.25	0	0	0.5
						L	112.5	0.625	0.6	1.8
10	Bulbar	81	Female	1	34	M	58.375	0	3.4	0.8
						C	115.75	0.375	6.1	4
						T	31	0	0.8	1.3
						L	151.125	0.25	9.1	6.3
11	Arm	70	Female	3	5	M	49.25	0	0	0
						C	81.5	0	0.6	0.4
						T	24.375	0	0	0
						L	139.125	0	0.8	0
12	Arm	61	Male	2.5	16	M	46.625	0	8.4	0.3
						C	79.75	0.25	16.1	0.4
						T	19.5	0	1.6	0.1
						L	101.375	0	1.7	3.4
13	Arm	55	Male	2	17	M	77.625	0	1	3.2
						C	45	0	1.3	1.8
						T	44.75	0	0.6	0.9
						L	96.25	0.625	4.3	10.2
14	Arm	54	Male	6.5	33	M	29.375	0	0.7	1.7
						C	34.75	0	0	0
						T	18.25	0	0	0
						L	91.875	0	0.1	0
15	Arm	68	Male	2.5	63	M	30.375	0.25	3	4.9
						C	47.625	0.25	1.8	5.4
						T	8.75	0.25	0.7	1
						L	102	1	1.9	9.7
16	Trunk	84	Male	1.2	21	M	50.375	0	0.2	0.4
						C	38.25	0	0.2	0.3
						T	29.875	0	0.2	0.3
						L	153.75	0	0.1	0
	Onset			Disease duration			Neuron			
17	Trunk	72	Male	3.25	22	M	66.5	0	0.1	0.4
						C	103	0	0.5	0.1
						T	26.75	0	0	0
						L	175.625	0	0.5	0
18	Trunk	73	Male	2.5	30	M	55	0	3.1	0.6
						C	101	1.125	8.3	4.5
						T	21.125	0	0.8	0
						L	111	0.5	7.6	2.2
19	Trunk	71	Male	1.5	32	M	52.25	0	1.9	1
						C	107.875	0.625	16.5	8.9
						T	22.875	0	4	1.1
						L	142.5	0.75	19.8	12.4
20	Trunk	74	Male	1.75	43	M	58	0	0.1	0.3
						C	90.75	0	9.5	2.4
						T	11.625	0	0.3	0.7
						L	89.25	0.875	7	2.5
21	Leg	77	Female	3	29	M	47.25	0	8	1.7
						C	81.25	0.25	8.8	2.4
						T	20.5	0	2.5	0.4
						L	85.375	0.875	7.9	3.9
22	Leg	73	Male	1	36	M	20.375	0	0	0
						C	135.125	0.125	4.1	1.8
						T	24.375	0	0	0
						L	129.625	0	0.7	0.3
23	Leg	81	Male	.75	41	M	63.75	0	7.8	1.6
						C	56.875	0	3	1.2
						T	23.5	0.125	2.4	0.5
						L	120.75	1	13.6	3.1
24	Leg	69	Female	5	45	M	37.25	0.125	3.8	1.4
						C	56.375	0.25	4	1.2
						T	15.875	0	0.5	0
						L	66.875	0.625	1.3	1.1
25	Leg	72	Female	1.5	68	M	n/a§	n/a§	n/a§	n/a§
						C	91.125	0.5	6.7	3.7
						T	29.625	0.125	0.1	0.3
						L	103	1.375	4.6	2.4

^*^Age at time of death. ^†^M: medulla; C: cervical cord;T: thoracic cord; L: lumbar cord. ^‡^ Numbers are averages of five observers. ^§^Region unavailable.

**Figure 2.**
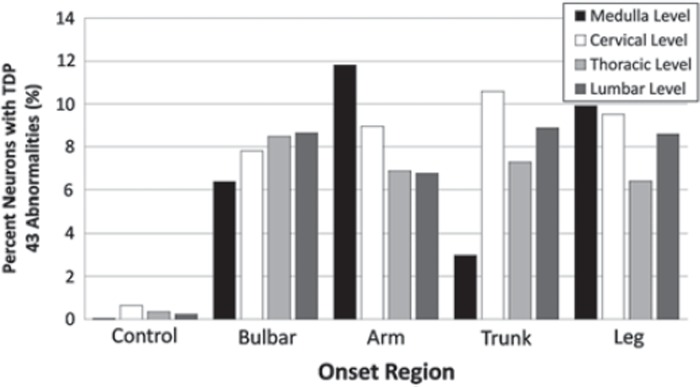
Bar graph showing the percentage of TDP-43 immunoreactive cytoplasmic aggregates at the cervical, thoracic, and lumbar spinal cord levels and the medulla for all 25 nervous systems. TDP-43 aggregates show no statistical correlation between frequency and anatomical distance from disease onset, and are not indexed to topographic advance of disease. Bar graph shows composite data.

At each neuraxis level, there was marked inter-but not intra-nervous system variability of frequency of TDP-43 aggregates (Table II). All four onset groups clustered close to the 8.3% TDP-43 aggregate average (bulbar onset average was 8.4%; arm onset average, 7.8%; trunk onset average, 8.1%; and leg onset average, 9.3%). The distribution of abnormalities within each nervous system did not correlate with the onset site and no topographic spread was apparent – this was sought both by total number of neurons showing TDP-43 aggregates and percentages of neurons showing aggregates at each neuraxis level – and thus neuraxis levels anatomically proximate to site of onset did not show statistical increase or decrease in aggregation relative to more remote sites (composite data are shown in Figure 2). There was no clear correlation between frequency of TDP-43 aggregates in nervous systems and clinical course, gender, or age of patients.

**Table II.** Percentage of ALS neurons with TDP-43 aggregates.

Level	Average	Range
Medulla	7.90%	0-26.8%
Cervical	9.70%	0-24.1%
Thoracic	6.60%	0-22.3%
Lumbar	8.00%	0-23.1%

**Figure 3.**
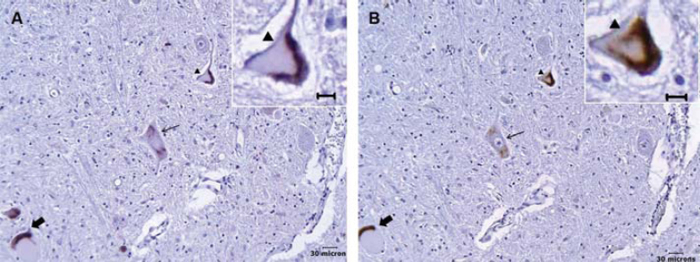
Correlation between ubiquitinated and TDP-43 immunoreactive cytoplasmic aggregates in ALS lower motor neurons. (A) Ubiquitinated aggregates in three separate motor neurons. (B) TDP-43 immunoreactive aggregates in the adjacent section showing corresponding changes (respective arrows). The morphological appearances are similar between ubiquitinated and TDP-43 immunostaining; inlays show respective magnified images (arrowhead).

Definite 1:1 correlation between ubiquitin and TDP-43 immunoreactive cytoplasmic aggregates was seen in 106 of the 113 motor neurons (Figure 3). While TDP-43 was not seen in the remaining seven motor neurons, this could be accounted for by the 6-μm-thick offset in histological sectioning. No ubiquitin immunoreactivity was identified in control lumbar motor neurons.

## Discussion

We found relatively low frequency and highly variable degrees of TDP-43 immunoreactive cytoplasmic aggregates over the rostral-caudal expanse of the lower motor neuron columns between different SALS nervous systems, but fairly uniform distribution between neuraxis levels within individual nervous systems. Our average of 8.3% of neurons with TDP-43 aggregates is consistent with a recent quantitative study by Giordana et al. (10) who found such aggregates in about 12% of neurons. They also reported a high frequency of abnormal TDP-43 diffuse cytoplasmic staining, which we sought but did not see with any significance, a difference probably attributable to technical rather than biologic factors since they used a four-fold higher concentration of the same primary antibody that we used. Thus, both quantitative studies taken together support the idea that TDP-43 aggregates involve only a subset of neurons and are distributed widely through the neuraxis.

The progression of appearance of TDP-43 abnormalities from solubility to insolubility, from nucleus to cytoplasm, and from puncta to skeins to dense round inclusions is unclear. Sanelli et al. (15) proposed that skeins develop into dense round inclusions. Giordana et al. (10) suggested that these changes are preceded by a shift from soluble to insoluble granule formation. Mori et al. (16) suggested that abnormal aggregation might occur in two independent parallel tracts, one leading to skein formation and another leading to dense round inclusions; they based their ontogeny on morphology and immunoelectronmicroscopy, but they did not quantify the absolute and relative frequency of abnormalities and examined only one spinal cord level (lumbar). In our data, the most common morphology of the aggregates was an overlap of skeins and dense round inclusions, then dense round inclusions, and only rarely pure skeins. This suggests a possible stoichiochemical equilibrium of the aggregates, which likely progresses from skeins to dense round inclusions, the majority of time being spent in an overlapping configuration.

Interestingly, there seems to be a lack of obvious correlation between the presence or absence of TDP-43 immunoreactive cytoplasmic aggregates and the morphological state of the neuron; pathological aggregation coexists in otherwise healthy appearing neurons and often does not seem to affect or correlate with its morphological status. Also interesting is that TDP-43 immunoreactive cytoplasmic aggregates were seen in small, atrophic neurons as well as large otherwise healthier appearing neurons at the high microscopy magnifications that we employed in the study. By combining high power microscopy and immunohistochemical markers, we were able to identify that small, atrophic cells were indeed motor neurons. Morphometric studies of ALS have failed to show cell shrinkage or left-shift of frequency histograms of neuron size and it is generally believed that neuronal loss is due to apoptosis, a presumed but not proven explanation (17,18). However, morphometry relies on visualization of nuclei and nucleoli to ensure uniform counting in thin two-dimensional cross-sectional analyses of large three-dimensional structures such as neurons in the spinal cord column and creates a bias against pathologic cells that undergo shrinkage and changes of the nucleus. That we observed TDP-43 immunoreactive cytoplasmic aggregates in neurons without nuclei and nucleoli suggests traditional morphometry might not be sensitive to atrophic changes of motor neuros. This raises the possibility that neuronal atrophy rathar than apoptotic cell death may play a greater role than has been previously recognized and, In turn, that there may be important mechanisms of neuron failure in ALS other than apoptosis. More studies are required to delineate this further.

An important observation was that the locations of TDP-43 cytoplasmic aggregates were uniformly distributed throughout the nervous system and did not show any graded correlation with respect to clinical disease onset. Lack of correlation might be partly explained by high background noise: there is marked variation among normal nervous systems; our sample sizes were small; and our patients ranged in age, gender, and disease course as well as site of onset. A correlation might have existed earlier in the disease course but by the time of post mortem observation, the nervous system is so oversaturated by the degeneration that gradations are undetectable. However, it is also possible that the TDP-43 aggregates themselves are of less importance in disease pathogenesis than altered protein function, an idea supported by recent studies by Giordana et al. (10) and Wegorzewska et al. (11). Sumi et al. related the degree of TDP-43 immunoreactive cytoplasmic aggregates to the clinical course of disease and found that patients with a rapid disease course, defined as disease duration of 2.5 years or less, showed higher levels of TDP-43 aggregation than those with a longer course, but they only looked at one neuraxis level and had sample size comparable to ours (19). Our findings do not support theirs, showing instead wide scatter. Thus, overall distribution of TDP-43 cytoplasmic aggregates combined with the marked variation in frequency of TDP-43 aggregation between nervous systems remains enigmatic and this raises intriguing questions about the biology of TDP-43.

A robust correlation was seen between ubiquitin and TDP-43 immunoreactive cytoplasmic aggregates, consistent with the belief that TDP-43 is the prime ubiquitinated protein. Recent studies by Sanelli et al. (15), Sumi et al. (19), and Giordana et al. (10) have raised the question whether TDP-43 is not the only ubiquitinated protein in ALS. While we find strong correlations between TDP-43 and ubiquitin, our technique relied on signal amplification from adjacent tissue sections and thus we are not able to participate further in this debate although follow-up colocalization studies are anticipated.

## References

[CIT0001] Leigh PN, Anderton BH, Dodson A, Gallo JM, Swash M, Power DM (1988). Ubiquitin deposits in anterior horn cells in motor neuron disease. Neuroscience Letters.

[CIT0002] Neumann M, Sampathu DM, Kwong LK, Truax AC, Micsenyi MC, Chou TT (2006). Ubiquitinated TDP-43 in frontotemporal lobar degeneration and amyotrophic lateral sclerosis. Science.

[CIT0003] Arai T, Hasegawa M, Akiyama H, Ikeda K, Nonaka T, Mori H (2006). TDP-43 is a component of ubiquitin-positive tau-negative inclusions in frontotemporal lobar degeneration and amyotrophic lateral sclerosis. Biochemical and Biophysical Research Communications.

[CIT0004] Mackenzie IR, Bigio EH, Ince PG, Geser F, Neumann M, Cairns NJ (2007). Pathological TDP-43 distinguishes sporadic amyotrophic lateral sclerosis from amyotrophic lateral sclerosis with SOD1 mutations. Ann Neurol.

[CIT0005] Robertson J, Sanelli T, Xiao S, Yang W, Horne P, Hammond R (2007). Lack of TDP-43 abnormalities in mutant SOD1 transgenic mice shows disparity with ALS. Neurosci Lett.

[CIT0006] Sreedharan J, Blair IP, Tripathi VB, Hu X, Vance C, Rogelj B (2008). TDP-43 mutations in familial and sporadic amyotrophic lateral sclerosis. Science.

[CIT0007] Kabashi E, Valdmanis PN, Dion P, Spiegelman D, McConkey BJ, van de Velde C (2008). TARDBP mutations in individuals with sporadic and familial amyotrophic lateral sclerosis. Nat Genet.

[CIT0008] van Deerlin VM, Leverenz JB, Bekris LM, Bird TD, Yuan W, Elman LB (2008). TARDBP mutations in amyotrophic lateral sclerosis with TDP-43 neuropathology: a genetic and histopathological analysis. Lancet Neurol.

[CIT0009] Zhang YJ, Xu YF, Cook C, Gendron TF, Roettges P, Link CD (2009). Aberrant cleavage of TDP-43 enhances aggregation and cellular toxicity. Proc Nat Acad Sciences.

[CIT0010] Giordana MT, Piccinini M, Grifoni S, de Marco G, Vercellino M, Magistrello M (2009). TDP-43 redistribution is an early event in sporadic amyotrophic lateral sclerosis. Brain Pathology.

[CIT0011] Wegorzewskaa I, Bell I, Cairns NJ, Miller TM, Baloh RH (2009). TDP-43 mutant transgenic mice develop features of ALS and frontotemporal lobar degeneration. Proc Natl Acad Sci USA.

[CIT0012] Ravits J, Laurie P, Fan Y, Moore DH (2007). Implications of ALS focality: rostral-caudal distribution of lower motor neuron loss post mortem. Neurology.

[CIT0013] Brooks BR, Miller RG, Swash M, Munsat TL (2000). El Escorial revisited: revised criteria for the diagnosis of amyotrophic lateral sclerosis. Amyotroph Lateral Scler Other Motor Neuron Disord.

[CIT0014] Olszewski J, Baxter D (1982). Cytoarchitecture of the Human Brain Stem.

[CIT0015] Sanelli T, Xiao S, Horne P, Bilbao J, Zinman L, Robertson J (2007). Evidence that TDP-43 is not the major ubiquitinated target within the pathological inclusions of amyotrophic lateral sclerosis. J Neuropathol Exp Neurol.

[CIT0016] Mori F, Tanji K, Zhang HX, Nishihira Y, Tan CF, Takahashi H (2008). Maturation process of TDP-43 positive neuronal cytoplasmic inclusions in amyotrophic lateral sclerosis with and without dementia. Acta Neuropathol.

[CIT0017] Sathasivam S, Ince PG, Shaw PJ (2001). Apoptosis in amyotrophic lateral sclerosis: a review of the evidence. Neuropathol Appl Neurobiol.

[CIT0018] Guégan C, Przedborski S (2003). Programmed cell death in amyotrophic lateral sclerosis. J Clin Invest.

[CIT0019] Sumi H, Kato S, Mochimaru Y, Fujimura H, Etoh M, Sakoda S (2008). Nuclear TAR DNA binding protein 43 expression in spinal cord neurons correlates with the clinical course in amyotrophic lateral sclerosis. J Neuropathol Exp Neurol.

